# Energy Conservation in Absorption Refrigeration Cycles Using DES as a New Generation of Green Absorbents

**DOI:** 10.3390/e22040409

**Published:** 2020-04-03

**Authors:** Reza Haghbakhsh, Hamed Peyrovedin, Sona Raeissi, Ana Rita C. Duarte, Alireza Shariati

**Affiliations:** 1 School of Chemical and Petroleum Engineering, Shiraz University, Mollasadra Ave., Shiraz 71348-51154, Iran or r.haghbakhsh@fct.unl.pt (R.H.); hamed.peyro@yahoo.com (H.P.); raeissi@shirazu.ac.ir (S.R.); 2 LAQV, REQUIMTE, Departamento de Química da Faculdade de Ciências e Tecnologia, Universidade Nova de Lisboa, 2829-516 Caparica, Portugal; ard08968@fct.unl.pt

**Keywords:** green solvent, novel solvent, deep eutectic solvents, energy, refrigeration, CPA

## Abstract

Deep eutectic solvents (DESs) are emerging green solvents with very unique characteristics. Their contribution to atmospheric pollution is negligible, and they can be “designed” for desired properties. In this study, the feasibility of applying DESs (Reline, Ethaline, or Glyceline) as absorbents in absorption refrigeration cycles was investigated. The sophisticated cubic-plus-association (CPA) equation of state, considering the strong intermolecular interactions of such complex systems, was used to estimate the thermodynamic properties. At a fixed set of base case operating conditions, the coefficients of performance were calculated to be 0.705, 0.713, and 0.716 for Reline/water, Ethaline/water, and Glyceline/water systems, respectively, while the corresponding mass flow rate ratios were 33.73, 11.53, and 16.06, respectively. Furthermore, the optimum operating conditions of each system were estimated. To verify the feasibility, results were compared to literature systems, including LiBr/water and various ionic liquid/water systems. The results indicate that DES/water working fluids have the potential to be used in such cycles. Since DESs have the characteristic to be tuned (designed) to desired properties, including their solvent power and their enthalpies of absorption, much further research needs to be done to propose new DESs with higher energy efficiencies.

## 1. Introduction

In the recent decades, the increasing amount of energy consumption and its consequences on the environment have become issues that must be seriously dealt with. The situation is so urgent that attention must be given to numerous parallel and complementing actions, in all aspects of environmental pollution control. Therefore, apart from the use of renewable energies, researchers have proposed various strategies for energy reduction and pollution control in the different industries and even for domestic demands [1,2,3,4,5,6]. The workhorses of many industries are the solvents, which unfortunately often release huge amounts of volatile organic compounds into the atmosphere. This results in damage to the ozone layer, global warming, and the degradation of fauna and flora, just to name a few. To reduce these harmful impacts, researchers are proposing novel green solvents to replace the conventional polluting solvents. Green solvents have the potential to be used in a wide range of applications, including refrigeration.

Refrigerators, which are indispensable industrial and domestic appliances, are actually among the highest energy-consuming equipment [7]. With the increasing concerns regarding energy consumption, absorption refrigeration systems have attracted more attention due to their superiority over conventional refrigeration cycles from an energy-saving point of view [7]. The main difference between absorption refrigeration and conventional vapor-compression cycles is in the compression mechanism, where the compression unit is replaced by an absorber/regenerator section [8], in which the working fluids are a refrigerant and an absorbent. 

By considering water as one of the working fluids, lithium bromide/water and water/ammonia are the two most-commonly used working fluids of absorption refrigeration systems in the literature [3,6,7,8,9,10]. However, the LiBr/water-based refrigeration systems have various issues, such as corrosion and crystallization, resulting in excessive maintenance costs, thus making them rather expensive [11,12]. On the other hand, water/ammonia solutions have health and environmental concerns [12,13]. These issues have led researchers to search for other working fluids for absorption refrigeration systems. 

Ionic liquids (ILs) are among the candidates to replace conventional absorbents because they possess various favorable characteristics, such as tunable properties, low vapor pressures, good chemical and thermal stabilities, acceptable solubilities, and non-flammability [6,10,11,12,13,14,15]. There are a number of studies that have applied ILs as absorbents in different absorption refrigeration systems [6,10,11,12,13,14,15]. However, ILs also have their own issues such as their high price and their corrosiveness, which result in limitations for their use as working fluids [16,17,18]. 

A new class of green solvents, called deep eutectic solvents (DESs), which have the potential to find their way into various industries, were introduced in 2004 by Abbott et al. [19]. A DES is actually an association mixture of at least two components (a hydrogen bond acceptor, HBA, and a hydrogen bond donor, HBD), which form a eutectic mixture by establishing hydrogen bonds. Because of the established hydrogen bonds between the HBA and HBD, the melting point of the mixture (DES) is lower than the melting points of the individual pure components. Due to these strong associations, DESs have most of the advantages of ILs, while overcoming some of their disadvantages [16,17]. They are prepared from cheaper compounds than ILs, and their preparation procedure is much easier. Therefore, DESs are much less costly than ILs [16,17]. Most DESs are also sustainable. Furthermore, since they are mostly nontoxic and have other favorable properties, such as acceptable solvating powers and low vapor pressures, research in the field is growing with a multitude of potential applications in mind [17,20,21,22,23,24,25,26,27].

However, with respect to the idea of utilizing a DES as an absorbent in absorption refrigeration systems, there is only one published study in the literature [5]. In that study, computational tools and molecular dynamic simulations were used to evaluate the application of either Reline, Ethaline, or Glyceline as the absorbent, with R134a as the refrigerant in an absorption refrigeration system [5]. It was shown that the working pair of Ethaline/R134a had the highest efficiency, while Reline/R134a showed the lowest efficiency [5]. The effects of the operational conditions on the efficiency of the absorption refrigeration cycle were not studied. 

Due to the highly desirable characteristics of DESs, the idea of using such novel solvents in absorption refrigeration cycles to replace the conventional solvents deserves further attention. Therefore, it is vital to have comprehensive investigations on different DESs for absorption refrigeration cycles and in combination with various refrigerants. In this work, the feasibility of using either Reline (1 choline chloride + 2 urea), Ethaline (1 choline chloride + 2 ethylene glycol), or Glyceline (1 choline chloride + 2 glycerol) as the absorbent and water as the refrigerant of the absorption refrigeration cycle was investigated by simulating the process and calculating the efficiencies. To obtain more accurate results, the thermodynamic properties of the pure refrigerant and the absorbent/refrigerant solutions were modeled using a sophisticated equation of state, which can handle the complexities of associating compounds. 

## 2. Methods 

### 2.1. Selected Deep Eutectic Solvents

Since DESs are novel and their physical data are quite scare, such feasibility studies are limited to only those DESs for which the basic information required by the thermodynamic models are available. By considering the very scarce literature data, ultimately Reline (1 choline chloride + 2 urea), Ethaline (1 choline chloride + 2 ethylene glycol), and Glyceline (1 choline chloride + 2 glycerol) were selected as the absorbents, and water was chosen as the refrigerant in the absorption refrigeration cycle. Table 1 presents some of the important physical properties of the investigated DESs in this study [28,29,30,31].

### 2.2. Absorption Refrigeration Cycle 

**Figure 1:**
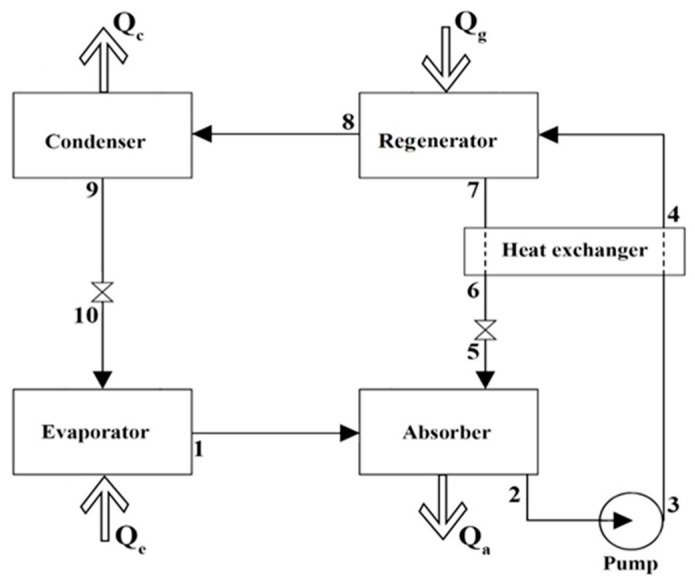
The schematic diagram of the absorption refrigeration cycle.

The schematic presentation of the DES/water absorption refrigeration cycle is shown in Figure 1. In the absorption refrigeration cycle, pure refrigerant (water) absorbs heat in the evaporator, *Q_e_*, then the evaporated refrigerant enters an absorber in which the non-volatile absorbent (DES) absorbs the refrigerant by discarding *Q_a_* to the surroundings. The resulting absorbent/refrigerant solution is pumped to the regenerator where the refrigerant is evaporated by adding heat *Q_g_* to the absorbent/refrigerant solution; however, because of its very low vapor pressure, the absorbent is not.

After separation, the pure refrigerant is led to a condenser where it is condensed by discarding *Q_c_* to the surroundings. The pressure on this condensate is reduced using a pressure reduction valve, which cools the refrigerant by the Joule–Thomson effect. The refrigerant then enters the evaporator once more to absorb heat, and the cycle is repeated. The remaining absorbent/refrigerant solution in the regenerator, with a lower refrigerant concentration, is recycled after exchanging heat in the heat exchanger where it is cooled by the absorbent/refrigerant solution being transported from the absorber to the regenerator. Its pressure is also reduced with the aid of a pressure reduction valve. The performance of the cycle can be evaluated by the coefficient of performance (COP), which is defined as

(1) $$COP=\frac{Q_{e}}{Q_{g}}$$

where $Q_{e}$ and $Q_{g}$ are the amounts of heat transferred in the evaporator and regenerator, respectively [9]. The value of the COP is calculated based on energy and mass balances [9]. Equations (2) and (3) give the absorbent mass balance and overall mass balance around the absorber unit, respectively.

(2) $$x_{5}m_{5}=x_{2}m_{2}$$

(3) $$m_{5}=m_{2}-m_{1}$$

in which *m_2_, m_1_,* and *m_5_* are the mass flow rates of the solution leaving the absorber, the pure refrigerant leaving the evaporator, and the solution entering the absorber, respectively, and *x_i_* is the mass fraction of the absorbent in stream *i*. By combining Equations (2) and (3),

(4) $$x_{5}(m_{2}-m_{1})=x_{2}m_{2}$$

The mass flow rate ratio, *f*, is defined as the ratio of the mass flow rate of the solution (absorbent + refrigerant) leaving the absorber ($m_{2}$) to the mass flow rate of the pure refrigerant entering the absorber ($m_{1}$),

(5) $$f=\frac{m_{2}}{m_{1}}$$

By using Equation (4), the mass flow rate ratio can be expressed as a function of the absorbent mass fraction in streams 5 and 2, as follows,

(6) $$f=\frac{m_{2}}{m_{1}}=\frac{x_{5}}{x_{5}-x_{2}}$$

By implementing the overall energy balance of the cycle, the COP of the absorption refrigeration cycle is derived as a function of mass flow rate ratio and the specific enthalpies in the form of Equation (7) [9],

(7) $$COP=\frac{Q_{e}}{Q_{g}}=\frac{h_{1}-h_{9}}{h_{8}+h_{5}(f-1)-h_{2}f}$$

where *h_i_* is the specific enthalpy of stream *i*. According to Equation (7), in order to calculate the COP of the cycle, the mass flow rate ratio and the values of the specific enthalpies of each stream are required. The specific enthalpy of each stream is a function of temperature, pressure, and mass fraction of each component in the stream, which can be calculated using the thermodynamic models [32] given in the following section. 

### 2.3. Thermodynamic Calculations 

#### 2.3.1. Specific Enthalpy 

The specific enthalpy of a pure component can be calculated by Equation (8) [33].

(8) $$h_{i}(T,P)=-RT^{2}{\left(\frac{\partial ln\varphi_{i}(T,P)}{\partial T}\right)}_{P}+h_{i}{}^{ig}(T)$$

which can be modified for the calculation of the enthalpies of multi-component mixtures as [33]

(9) $$h(T,P,x)=-RT^{2}\sum_{i=1}^{N}x_{i}{\left(\frac{\partial ln\varphi_{i}(T,P,x)}{\partial T}\right)}_{P,x}+\sum_{i=1}^{N}x_{i}h_{i}{}^{ig}(T)$$

where *φ_i_* is the fugacity coefficient, *h_i_^ig^* is the ideal gas enthalpy of component *i*, and *N* is the number of components in the mixture.

The ideal gas enthalpy, *h_i_^ig^*, is calculated by Equation (10).

(10) $$h_{i}^{ig}=\int C_{p_{i}}^{ig}dT$$

where *C_pi_^ig^* is the ideal gas heat capacity of component *i*, with the formulation given by Equation (11) for water and the three investigated DESs.

(11) $$C_{p_{i}}^{ig}=A_{i}+B_{i}T+C_{i}T^{2}+D_{i}T^{3}$$

In Equation (11), *A_i_* to *D_i_* are the constants for component “*i*”, which were calculated for the investigated DESs of this study based on the Joback group contribution method [34]. The resulting values for the three deep eutectic solvents, as well as water [35], are presented in Appendix A. 

The fugacity coefficients, *φ*, in Equations (8) and (9) of the pure components and the multicomponent mixtures (DES/water) were calculated based on the equation of state approach, which is explained in the next section. 

#### 2.3.2. The Cubic-Plus-Association Equation of State

In order to calculate the fugacity coefficients, as well as the unknown temperatures and pressures, which are thermodynamically interrelated to the design temperatures and pressures of the cycle, it is necessary to use a powerful and accurate thermodynamic model, such as an equation of state. However, DESs are highly associating mixtures, and hence, conventional cubic equations of state are not suitable thermodynamic models for such systems involving hydrogen bonds. The cubic-plus-association equation of state (CPA EoS) is among the most powerful equations of state that can handle associating compounds. Because of this, the CPA has been used in different studies involving physical property estimations or phase equilibrium calculations of DESs and their mixtures [36,37,38,39]. Viscosity, which is a challenging thermophysical property to model, was modeled successfully for a range of DES families using the CPA EoS coupled to theoretical models such as the free volume or the friction theory [36,38]. In other studies, the solubilities of CO_2_ and SO_2_ in different types of DESs were modeled successfully using the CPA EoS [37,38].

CPA is a five-parameter equation of state, which is actually a combination of the two parts of physical interactions and associations and is presented as [40,41],

(12) $$P=\frac{RT}{v-b}-\frac{a}{v(v+b)}-\frac{1}{2}\frac{RT}{v}(1+\rho \frac{\partial \ln g(\rho )}{\partial \rho })\sum_{j}^{}x_{j}\sum_{A_{j}}^{}\left(1-X_{A_{j}}\right)$$

where b is the co-volume parameter, v is the molar volume, and *a* is the energy parameter, which is defined by [40],

(13) $$a(T)=a_{0}{\left[1+c_{1}(1-\sqrt{T_{r}})\right]}^{2}$$

In this equation, $T_{r}$ is the reduced temperature, and $a_{0}$ and $c_{1}$ are constants. In Equation (12), g(ρ) is the radial distribution function, which is defined as follows [42],

(14) $$g(\rho )=\frac{1}{1-1.9(\frac{b\rho }{4})}$$

In Equation (12), $X_{A_{j}}$ is the fraction of *A*-sites of molecule *j* that are not bonded to other substances. This parameter is calculated using Equations (15) and (16) [43,44,45].

(15) $$X_{A_{j}}=\frac{1}{1+\rho \sum_{i}^{}x_{i}\sum_{B_{i}}^{}X_{B_{i}}\Delta^{A_{j}B_{i}}}$$

and

(16) $$\Delta^{A_{j}B_{i}}=g(\rho )(\exp(\frac{\varepsilon^{A_{j}B_{i}}}{RT})-1)b_{ji}\beta^{A_{j}B_{i}}$$

where $\Delta^{A_{j}B_{i}}$ is the association strength, $\varepsilon^{A_{j}B_{i}}$ is the association energy, and $\beta^{A_{j}B_{i}}$ is the association volume parameter of the interactions between sites *A_j_* and *B_i_*. In general, the CPA has five parameters that are optimized for each compound in its pure state: *a_0_, b, c_1_*, $\varepsilon^{A_{j}B_{i}}$, and $\beta^{A_{j}B_{i}}$. Based on Equation (15), $X_{A_{j}}$ depends on the hydrogen-bonding scheme of the component. Huang and Radosz introduced eight types of hydrogen-bond schemes that are used for the calculation of $X_{A_{j}}$ [46]. They categorized a number of compounds within general groups such as water, alcohols, amines, glycols, acids, etc., proposing specific association schemes for each [46]. The pseudo-pure component approach is commonly used for DESs, meaning that the combination of the HBA and HBD in the mixture is considered as one pseudo-component [36,37,38,39]. For the DES pseudo-component, the association scheme of 2B [46] is considered, while water is represented by the 4C scheme [37,47,48]. This means that a water molecule has four sites on its molecule for possible hydrogen bonding, while a DES has the potential for making hydrogen bonds at two sites. 

In order to extend the CPA to mixtures, the following mixing and combining rules [47] are applied,

(17) $$a(T)=\sum_{i}^{}\sum_{j}^{}x_{i}x_{j}a_{ij}$$

(18) $$a_{ij}=\sqrt{a_{i}a_{j}}(1-k_{ij})$$

(19) $$b=\sum_{i}^{}x_{i}b_{i}$$

(20) $$b_{ij}=\frac{b_{i}+b_{j}}{2}$$

(21) $$\varepsilon^{A_{j}B_{i}}=\frac{\varepsilon^{A_{j}B_{j}}+\varepsilon^{A_{i}B_{i}}}{2}$$

(22) $$\beta^{A_{j}B_{i}}=\sqrt{\beta^{A_{j}B_{j}}\beta^{A_{i}B_{i}}}$$

where *k_ij_* in Equation (18) is the binary interaction parameter, which is considered as follows,

(23) $$k_{ij}=k_{0}T+k_{1}$$

$k_{0}$ and $k_{1}$ are adjustable parameters, which are optimized based on the experimental data [37]. 

After determining the pure CPA parameters and optimizing the binary interaction parameters for the desired system, the fugacity coefficients of all the components in all of the streams of Figure 1 can be calculated using Equation (24),

(24) $$RT\ln\varphi_{i}=\int_{V}^{\infty }\left[{\left(\frac{\partial P}{\partial n_{i}}\right)}_{T,V,n_{j}}-\frac{RT}{V}\right]dV-RT\ln(\frac{Pv}{RT})$$

### 2.4. Simulation of the Process 

According to the above-mentioned equations for enthalpy calculations, and with the aid of the CPA model, the enthalpy of each stream is calculated. With the resulting values, the COP of the system can be estimated. However, to carry out the simulation, a number of commonly considered assumptions are applied as follows [9]: (1) The process is steady state; (2) pressure losses in the connecting pipes and the units are neglected; (3) streams 1, 8, 9, and 10 are considered to be pure refrigerant; (4) thermodynamic equilibrium is assumed in the evaporator, absorber, regenerator, and condenser; (5) the refrigerant in stream 8 is superheated vapor at the regenerator temperature and condenser pressure; (6) the expansion process from stream 9 to 10 is considered isenthalpic; and (7) the heat exchanger efficiency is 100%.

The simulation is carried out in the following manner: The condenser and evaporator pressures are first determined. According to assumptions (3) and (4), the condenser and evaporator pressures are equal to the vapor pressures of the pure refrigerant at the condenser and evaporator temperatures, respectively. Based on assumption (5), the enthalpy of stream 8 is calculated directly at the corresponding temperature and pressure. To calculate the enthalpy of the solutions of streams 2 and 5, in addition to the temperature and pressure, the mass fractions of each component are required. The temperature of stream 5 is estimated through bubble point temperature calculations with the aid of the CPA EoS, *x_2_* and *x_7_* are calculated through flash calculations using CPA, and finally, *x_5_*=*x_7_*. All calculations are carried out on the basis of a refrigerant mass flow rate of 1 kg/s. In this manner, the enthalpies of all of the required streams and the mass flow rate ratio of the cycle are determined, allowing one to calculate the COP of the system using Equation (7). 

## 3. Results and Discussion 

### 3.1. CPA Equation of State Parameterization

Table 2 presents the calculated values of the critical properties of the investigated DESs based on the method of Valderrama and Rojas [37,49], as well as for water [8]. Table 2 also presents the values of the CPA parameters for each of the investigated DESs and water as reported in the literature [37,47]. 

In order to use the CPA EoS for the mixtures of DES + water, it is necessary to find the interaction parameters between each DES and water. It is common practice to optimize the binary parameters of CPA using vapor–liquid equilibria of the desired mixture; however, this is not possible in the case of DESs, which have negligible vapor pressures. Therefore, the density data of the DES + water mixtures were used instead to optimize the interaction parameters between each DES and water. Table 3 presents the density data references for the mixtures of DES + water, as well as the ranges of pressures, temperatures, and compositions used in optimization [29,30,50,51,52,53,54]. The constants of Equation (23) were thus optimized using a genetic algorithm (GA), with the following objective function,

(25) $$OF=\sum_{i}^{N}(\frac{\rho_{i}^{\exp.}-\rho_{i}^{calc.}}{\rho_{i}^{\exp.}}{)}^{2}$$

where $\rho_{i}^{calc.}$ and $\rho_{i}^{\exp.}$ are the CPA-calculated and experimental liquid densities of the investigated DES + water mixtures, respectively, and *N* is the number of data points. The resulting optimized values of the constants *k_0_* and *k_1_* in Equation (23) are presented in Table 3.

### 3.2. Base Case

In order to have a fair comparison of the COPs of the absorption refrigeration cycles in this study to those of the different working pairs of the literature, a “base case” was considered at the operating conditions commonly considered for many of the literature studies in which water was used as the refrigerant [9,14]. In this base case, the condenser, evaporator, regenerator, and absorber temperatures were considered as 40, 10, 100, and 30 °C, respectively. At these set temperatures, the prevailing pressures in the condenser and evaporator are 7.38 and 1.23 kPa, respectively [8]. The resulting COPs of the absorption refrigeration cycle for each of the investigated DES/water working pairs are reported in Table 4 for the base case. In this table, a comparison is also provided with other literature absorption refrigeration cycles for various working pairs when water was used as the refrigerant at the same base case condenser, evaporator, regenerator, and absorber temperatures [14]. Additionally, the concentrations of solvent (mass %), temperatures, and pressures of the DES-rich and DES-lean solutions are presented in Appendix B. 

Table 4 indicates that the investigated DES/water working pairs have similar COPs to the conventional LiBr/water working pair but higher COPs than the absorption refrigeration cycles with the IL/water working pairs. 

Additionally, based on the energy balance over the evaporator,

(26) $$Q_{e}=h_{1}-h_{10}=h_{1}-h_{9}$$

where the amount of heat absorbed in the evaporator, $Q_{e}$, is a function of the enthalpies of the pure refrigerant ($h_{1}$ and $h_{9}$). Since water is the common refrigerant in all of the cycles considered, and the temperatures of the condenser and evaporator are fixed at the base case, these enthalpies ($h_{1}$ and $h_{9}$) are the same for all of the cycles presented in Table 4. Based on Equation (26) and with the aid of the CPA EoS, this constant base case value of $Q_{e}$ is found to be 2150.9 kW for all of the cycles. The corresponding literature value for IL/water systems [14] is 2354.49 kW. The difference is due to the different procedures of calculating the enthalpies.

With a constant value of $Q_{e}$ for all of the investigated cycles, based on Equation (1), only $Q_{g}$ affects the value of COP, through an inversely proportional relationship. Based on the energy balance (Equation (27)) and using the CPA EoS to calculate the required enthalpies, $Q_{g}$ is calculated and presented in Table 4.

(27) $$Q_{g}=h_{8}+h_{5}(f-1)-h_{2}f$$

According to the results, the input heat to the generator to separate the absorbent from the refrigerant is lower for the DES/water cycles than for the IL/water cycles, while it is similar to that of the LiBr/water cycle. The differences between the $Q_{g}$ values, and hence the COPs of the presented cycles, are due to the differences in both the water solubilities in the absorbents and the enthalpies of the absorbent/water solutions. 

Apart from the considerations of energy, compared for the different cycles through COP, the mass flow rates within the cycles are also of great significance in the final selection of the most economic cycle. The calculated and reported values of mass flow rate ratios are also presented in Table 4. The differences among the cycles are due to the different solubilities of the refrigerant in the absorbent in the regeneration and the absorber units. According to Equation (6), the mass flow rate ratio is a function of *x_5_* and *x_2_,* which is also given in Table 4 for each cycle. The mass flow rate ratios of the DES/water working pairs are mostly in the same order of magnitude as those of IL/water working pairs, while being about 3 to 8 times that of the LiBr/water system. Higher mass flow rate ratios require greater absorbent/refrigerant flow rates, which result in increased pumping costs of the working fluid. 

By comparing the three studied DESs among themselves, the Reline/water cycle has the highest value of $Q_{g}$, which leads the lowest COP. At the base case conditions, it can be seen that Reline has the highest water solubilities in the absorber and regenerator, yet the Reline/water system has the highest mass flow rate ratio as well. Based on Equation (6), the mass flow rate ratio is also an inverse function of the difference between the solubilities of water in the absorber and the regenerator, which has the lowest value for the Reline/water system (2.89%) among the studied DES/water systems, and thus the highest mass flow rate ratio. 

With an economical perspective, a quantitative comparison of the DES/water mass flow rate ratios indicates the values to vary from 11.53 for Ethaline/water to 33.73 for Reline/water. On the other hand, $Q_{g}$ values do not differ significantly at the base case conditions. Based on these results, and the fact that $Q_{g}$, and consequently COP, are functions of both enthalpy and water solubility, it is suggested that the effects of the enthalpy values on $Q_{g}$ and COP are more important than the effects of mass flow rate ratio on $Q_{g}$ and COP. 

From an overall perspective, a comparison of the COP values of the three DES/water working pairs to that of the traditional LiBr/water cycle shows that all three DESs, especially Ethaline/water, can be applicable as absorbents in absorption refrigeration cycles, while they are even better than the ILs investigated in the literature (Table 4) regarding the point of view of COP. On the other hand, the DES systems have higher mass flow ratios than LiBr/water. Still, they may have the potential to replace LiBr/water because the latter is associated with different problems such as corrosion, crystallization, and high maintenance costs. Even if the advantages of the three investigated DESs are not significant, it should be noted that numerous new DESs will be on the way, and since the properties of DESs can be tuned, it is very likely that upon the availability of experimental data on new DESs, some will have the thermodynamic properties to result in paramount improvements in absorption refrigeration efficiencies. Thus, this new class of solvents do indeed deserve further investigation, and it is very well possible that in the future, they can replace the working pairs of today.

Furthermore, in order to have a general view of the COP values of DES systems with respect to that of an ideal absorption refrigeration cycle, Equation (28) estimates the highest possible COP for an ideal cycle [55],

(28) $$COP_{ideal}=(\frac{T_{G}-T_{A}}{T_{G}})(\frac{T_{E}}{T_{C}-T_{E}})$$

According to Equation (28), the ideal COP at the base case conditions was calculated to be 1.77. The COP values of the three DES/water cycles are much lower than the ideal cycle, which is of course to be expected, and so, the results seem reasonable.

In addition to thermodynamic and energetic analyses, the viscosities of the working pairs are also among the operational properties of concern in these cycles, which should be taken into consideration. As an example, by considering the conditions of stream 2 (outlet of the absorber) based on studies from the literature [56,57], the viscosities of Reline/water, Ethaline/water, Glyceline/water, and LiBr/water solutions are 275, 14.8, 81.7, and 3.7 mPa.s, respectively. The viscosities are higher for all three DES/water working pairs as compared to the LiBr/water working pair, especially for Reline/water and Glyceline/water, which are significantly higher. 

### 3.3. The Effect of Regenerator and Absorber Temperatures 

**Figure 2:**
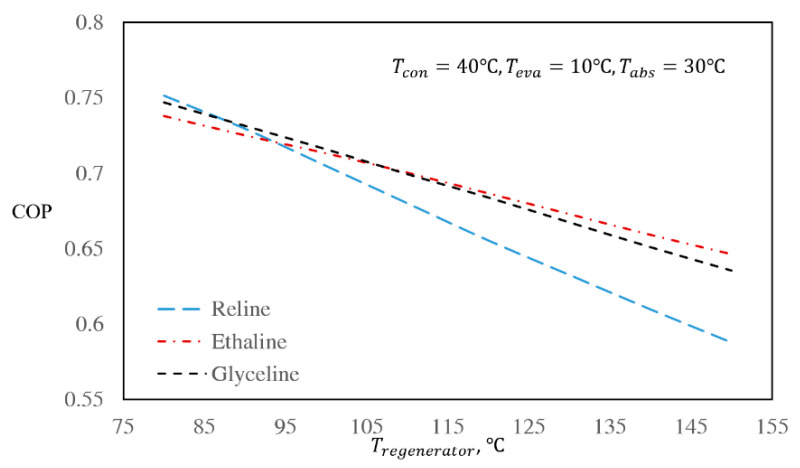
Effect of regenerator temperature on the absorption refrigeration coefficients of performance (COPs) of the investigated DES/water working pairs for the base case.

**Figure 3:**
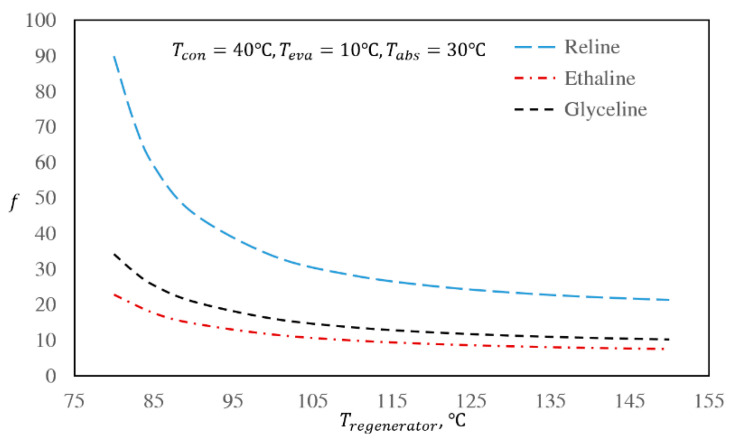
Effect of regenerator temperature on the absorption refrigeration flow rate ratios of the investigated DES/water working pairs for the base case.

Due to the importance of the regenerator and absorber sections, it is useful to investigate the behavior of the DES/water cycles at various regenerator and absorber temperatures [9]. The effect of the regenerator temperature on the COP is presented in Figure 2, while Figure 3 shows the effect of the regenerator temperature on the mass flow rate ratio. In these figures, the condenser, evaporator, and absorber temperatures were kept at the base case conditions, i.e., at 40, 10, and 30 °C, respectively.

According to Figure 2, by increasing the regenerator temperature, the COP decreases in all three systems. Based on the energy balance over the evaporator, which is presented as Equation (26), the amount of heat, which is absorbed by the evaporator,$Q_{e}$, is a function of the enthalpies of the pure refrigerant and is dependent on the condenser and evaporator temperatures. However, it is independent of the regenerator and absorber temperatures. Therefore, based on Equation (1), only $Q_{g}$ affects the COP. By increasing the regenerator temperature, the solubility of the refrigerant in the absorbent decreases, which leads to a lower mass flow rate of the solution. However, the heat input to the regenerator increases [58], thus the COP decreases due to the increased regenerator temperatures, as shown in Figure 2. 

Based on Figure 3, the behavior of mass flow rate ratio, *f*, against regenerator temperature is not linear. This is due to the definition of *f*. Based on Equation (6), when the difference between the values of *x_5_* and *x_2_* decreases, the mass flow rate ratio increases nonlinearly. In absorption refrigeration cycles, the regenerator pressure is always higher than the absorber pressure by the amount generated by the pump. It is expected that when the pressure is higher, the solubility of the refrigerant in the absorbent is greater. Accordingly, in the regenerator, heat should be added to the system to increase the absorbent/refrigerant solution temperature. By increasing the regenerator temperature, the vapor pressure of the refrigerant is increased, and therefore, separation of the refrigerant from the solution occurs. By increasing the amount of heat input to the regenerator, higher amounts of refrigerant can be separated from the solution. If enough heat is added to the regenerator, the refrigerant leaves the solution in a way that the mass fraction of the absorbent in the outlet solution from the regenerator (*x_5_*) is greater than that of the inlet solution to the regenerator (*x_2_*). When all temperatures are kept constant and only the regenerator temperature is increased, *x_5_* increases accordingly. On the other hand, *x_2_* remains constant, since it is only a function of absorber temperature and pressure. Therefore, the difference between *x_5_* and *x_2_* increases, leading to decreased values of *f*, as can be seen in Figure 3. 

**Figure 4:**
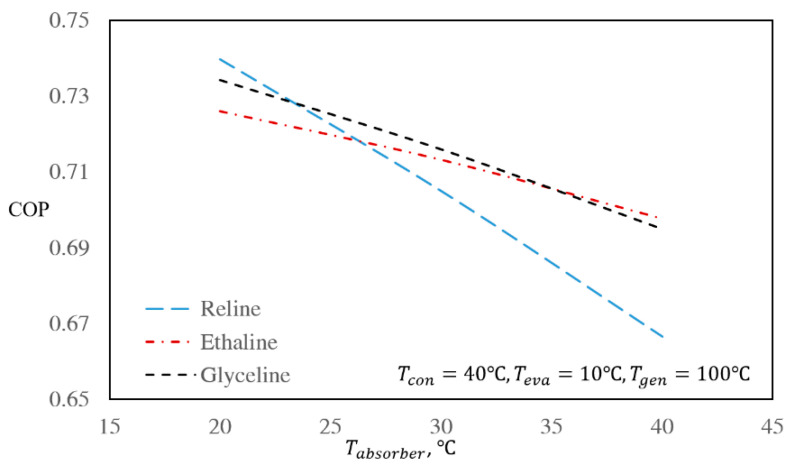
Effect of absorber temperature on the absorption refrigeration COPs of the investigated DES/water working pairs for the base case.

**Figure 5:**
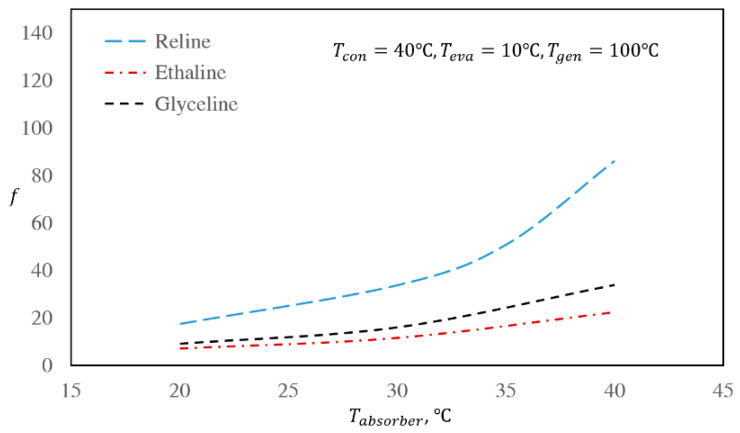
Effect of absorber temperature on the absorption refrigeration flow rate ratios of the investigated DES/water working pairs for the base case.

Figure 4 and Figure 5 present the effect of absorber temperature on the COP and mass flow rate ratio of the cycle, respectively, while keeping the temperatures of the condenser, evaporator, and regenerator constant at 40, 10, and 100 °C, respectively. 

Based on Figure 4, by increasing the absorber temperature, the COP of the system decreases. At increased absorber temperatures, the solubility of the refrigerant in the absorbent decreases; hence, it is necessary to add greater amounts of the absorbent to absorb the required amount of refrigerant. In this way, the mass flow rate of the solution increases, and consequently, the regenerator heat, *Q_g_*, must be increased to adequately separate the refrigerant from the absorbent. Therefore, the COP of the system decreases. According to Figure 5, by increasing the absorber temperature, the value of *f* also increases. When the temperatures of all the other units are constant and only the absorber temperature is increased, *x_2_* increases due to increasing vapor pressures of the refrigerant, while *x_5_* remains constant because it is independent of absorber temperature. In this way, the difference between *x_5_* and *x_2_* decreases, which causes the *f* value to increase based on Equation (6). 

By comparing the mass flow rate ratios of the three investigated DES/water working pairs, it is seen that the mass flow rate ratio of the Glyceline/water working pair is lower than Reline/water and slightly higher than Ethaline/water. Based on Figure 4, as well as Figure 2, at low regenerator and absorber temperatures, the COP of the Reline/water working pair is higher than those of Ethaline/water and Glyceline/water. By increasing the regenerator and absorber temperatures, the COP of the Reline/water working pair decreases. The reason for this behavior is the differences between the ideal gas heat capacities and the water solubilities in the absorbents as functions of temperature. With this same reasoning, the COP of the Glyceline/water working pair at lower regenerator and absorber temperatures is higher than Ethaline/water, while at higher absorber and regenerator temperatures, the Glyceline/water COP becomes lower than Ethaline/water. 

In general, by considering Figure 2 and Figure 3, it can be seen that for all three DES/water systems within the same regenerator temperature range (80 to 150 °C), the changes in mass flow rate ratios are much higher than the changes in COPs. This suggests that the effect of enthalpy is more important on the COP value than mass flow rate ratio. The same behavior was seen in the base case conditions for all three DES/water systems, which demonstrates that in different absorption refrigeration cycles using the same refrigerant, the enthalpy of the working fluid is more important than the mass flow rate ratio. 

This conclusion is further confirmed by Figure 4 and Figure 5, where the mass flow rate ratio changes are far greater than the changes in the COP values for all three studied systems. 

### 3.4. Analysis by Experimental Design

In order to have a more comprehensive investigation, the effect of condenser, evaporator, absorber, and regenerator temperatures on the COP of the studied working fluids was investigated through experimental design. For this purpose, the COPs were calculated within wide ranges of condenser, evaporator, absorber, and regenerator temperatures (Table 5). 

Response surface methodology (RSM) [59] was used for experimental design, and 480 different tests were suggested by the experimental design for each DES/water working pair. All of the suggested tests were carried out and calculated by our model, and the values of the resulting COPs and mass flow rate ratios for each test were inserted into the experimental design analysis to develop the final model. Based on the results of the experimental design analyses, the standard deviation and the mean and *R*-Squared values of each system were calculated and are presented in Table 6.

Equations (29) to (31) are the final proposed models by experimental design analyses for each of the investigated DES/water working pair systems, quantifying the effects of temperature on the COP. The proposed correlations are only applicable for the given temperature ranges in Table 5 for each system. All of the other factors that affect the values of COP are considered to be constant. By using these equations, the values of COP for each system at different system conditions can be calculated.

(29) $$\begin{array}{l} COP_{Reline/water}=0.949+3.957\times 10^{-3}T_{eva}-2.571\times 10^{-3}T_{gen}-2.432\times 10^{-3}T_{abs}+2.300\times 10^{-4}T_{con}\\ +2.200\times 10^{-5}T_{eva}T_{gen}+6.200\times 10^{-5}T_{eva}T_{abs}-6.200\times 10^{-5}T_{eva}T_{con}-3.500\times 10^{-5}T_{gen}T_{abs}\\ +2.300\times 10^{-5}T_{gen}T_{con}+7.500\times 10^{-5}T_{abs}T_{con}-3.700\times 10^{-5}T_{eva}{}^{2}-2.200\times 10^{-5}T_{abs}{}^{2}-2.500\times 10^{-5}T_{con}{}^{2} \end{array}$$

(30) $$\begin{array}{l} COP_{Ethaline/water}=0.820+5.404\times 10^{-3}T_{eva}-1.607\times 10^{-3}T_{gen}+1.365\times 10^{-3}T_{abs}+5.00\times 10^{-4}T_{con}\\ +2.200\times 10^{-5}T_{eva}T_{gen}-3.900\times 10^{-5}T_{eva}T_{abs}-6.200\times 10^{-5}T_{eva}T_{con}-1.400\times 10^{-5}T_{gen}T_{abs}+2.400\times 10^{-5}T_{gen}T_{con}\\ +2.500\times 10^{-5}T_{abs}T_{con}-1.050\times 10^{-4}T_{eva}{}^{2}-3.900\times 10^{-5}T_{abs}{}^{2}-4.000\times 10^{-5}T_{con}{}^{2} \end{array}$$

(31) $$\begin{array}{l} COP_{Glycine/water}=0.898+2.028\times 10^{-3}T_{eva}-1.749\times 10^{-3}T_{gen}-9.120\times 10^{-4}T_{abs}-4.830\times 10^{-4}T_{con}\\ +1.800\times 10^{-5}T_{eva}T_{gen}+3.300\times 10^{-5}T_{eva}T_{abs}-4.600\times 10^{-5}T_{eva}T_{con}-2.900\times 10^{-5}T_{gen}T_{abs}+2.000\times 10^{-5}T_{gen}T_{con}\\ +6.200\times 10^{-5}T_{abs}T_{con}-1.400\times 10^{-5}T_{abs}{}^{2}-2.900\times 10^{-5}T_{con}{}^{2} \end{array}$$

Due to the importance of the regenerator and absorber sections, the optimum value of COP for each absorption refrigeration cycle and the corresponding operating conditions are calculated based on the final proposed model of experimental design and are presented in Table 7. To calculate the optimum COPs for the studied working fluids, the condenser and evaporator temperatures were set equal to the base case values, and only the regenerator and absorber temperatures were optimized. The maximum achieved COP belongs to the Reline/water working pair, having a value of 0.816. 

### 3.5. Effect of Pump Work

Studies in the literature on absorption refrigeration cycles have determined that the work done by the pump is negligible in comparison to the amount of heat transfer in the regenerator [7,9,10,60]. Accordingly, the work done by the pump, *W_p_*, was neglected in this work. However, to make sure that this is indeed a valid assumption, the work done for pumping all three DES/water working pairs was calculated using Equation (32) [60].

(32) $$W_{P}=\frac{(P_{3}-P_{2})V}{\eta_{p}}$$

where *V* is the volumetric flow rate of the solution, and *η_p_* is the efficiency of the pump. After determining the work done by the pump, the effect of this parameter on the calculations was examined by use of the heat ratio, *η*, which is defined as Equation (33) [9].

(33) $$\eta =\frac{Q_{e}}{Q_{g}+W_{P}}$$

Accordingly, the results of the calculations at the base case conditions are shown in Table 8.

Table 8 presents the effect of the pump work on the cycle. The required work for pumping is higher for the Reline/water solution than for the other two working pairs because of its higher mass flow rate. Based on the comparison, it can be seen that the work of the pump is less than 0.01% of the heat transferred in the regenerator for all three investigated working pairs. Accordingly, the results confirm that including the pump work does not have a significant effect on the calculations, and neglecting this term for simplification is an acceptable assumption.

## 4. Conclusions 

The feasibility of applying DESs to typical absorption refrigeration cycles was investigated. The CPA EoS was used to calculate the required thermodynamic properties, such as solubilities, enthalpies, bubble point temperatures, and equilibrium concentrations. The results indicate that at similar operating conditions, the investigated DES/water cycles have similar COP values but higher mass flow rate ratios than the conventional cycle of LiBr/water. 

Using experimental design analyses, the functionality of COP was determined, and the optimum operating conditions of the cycles were determined. Accordingly, the highest COPs for the Reline/water, Ethaline/water, and Glyceline/water systems were calculated to be 0.816, 0.776, and 0.786, respectively, and the mass flow rate ratios corresponding to the calculated maximum COPs were 244.3, 30.8, and 47.9, respectively. 

The results demonstrate that DES/water working fluids can have the potential to be used in absorption refrigeration cycles. Among the investigated DESs, the Ethaline/water working pair has the greatest potential because of its similar COP but significantly lower mass flow rate ratio compared to either Reline/water or Glyceline/water. Comparisons among novel solvents indicated that DESs have more potential than ionic liquids for use in absorption refrigeration cycles. This is inspiring because DESs are also easier to prepare and less costly than ionic liquids. However, the most promising aspect for future applications of DESs as absorbents lies in the tunability of these novel solvents. A vast range of DESs are possible by the engineered selection from a rich choice of HBAs and HBDs and at various molar ratios. Therefore, when experimental data become available on new DESs, there will be choices that have highly desired solubilities and enthalpies as working fluids, which will result in absorption refrigeration cycles with much better performances. 

## Figures and Tables

**Table 1:** The physical properties of the investigated deep eutectic solvents (DESs) in this work.

DES Name	HBD	HBA	HBA/HBD Molar Ratio	Mw	Freezing Point (K)	$\mathrm{Density}\;(\frac{g}{cm^{3}})\;\mathrm{at}$ 25 °Cand 0.1 MPa
Reline	Urea	Choline chloride	1:2	86.580	285.00 ^4^	1.19790 ^2^
Ethaline	Ethylene glycol	Choline chloride	1:2	87.920	207.14 ^1^	1.11704 ^3^
Glyceline	Glycerol	Choline chloride	1:2	107.933	271.82 ^1^	1.19123 ^3^

^1^ Reference [28]. ^2^ Reference [29]. ^3^ Reference [30]. ^4^ Reference [31].

**Table 2:** The cubic-plus-association (CPA) parameters for water [47] and the investigated DESs in this study [37], and the calculated values of the critical properties and acentric factors of the DESs.

DES	$a_{0}(\frac{bar.L^{2}}{mol^{-2}})$	$b(\frac{L}{mol})$	$C_{1}$	β	$\varepsilon (\frac{bar.L}{mol})$	*Tc (K)*	*Pc (MPa)*	*ω*
Reline ^1^	28.31	0.0657	0.116	0.5458	1001.70	644.44	5.0149	0.6167
Ethaline ^1^	29.28	0.0733	0.835	0.5456	1001.70	602.00	4.1661	0.8747
Glyceline ^1^	26.53	0.0829	0.810	0.5585	1018.30	680.67	3.4183	1.1780
Water ^2^	1.22777	0.0145	1.180	0.0250	140.36	647.10	22.055	0.345

^1^ The association scheme of 2B, the CPA parameters, and the critical properties were taken from [37]. ^2^ The association scheme of 4C, the CPA parameters, and the critical properties were taken from [47,48].

**Table 3:** The optimized binary interaction parameter constants and the range of experimental data used when optimizing (Equation (23)).

Solution	Temperature Range (K)	Pressure Range (MPa)	DES Mole Fraction Range	Density Range $\left(\frac{kg}{m^{3}}\right)$	Ndp	10^3^*k_0_*	10^5^*k_1_*	Ref
Reline/water	293.15–363.15	0.1–50	0–1	965.0–1205.8	682	−1.66406	−1.33	[29,50]
Ethaline/water	283.15–363.15	0.1–50	0–1	965.0–1130.6	781	−2.40793	−1.44	[30,51,52]
Glyceline/water	283.15–363.15	0.1–50	0–1	965.0–1202.4	808	−2.61677	-1.39	[30,53,54]

Ndp represents the number of data points.

**Table 4:** Comparison of the investigated absorption refrigeration cycle working pairs in this study with literature working pairs when water is used as the refrigerant.

Working Pair	COP	*f*	*x_5_* Mass%	*x_2_* Mass%	*Q_g_* (kW)
Reline/water	0.705	33.73	97.46	94.57	3052.35
Ethaline/water	0.713	11.53	92.38	84.37	3017.36
Glyceline/water	0.716	16.06	94.50	88.61	3004.06
LiBr/water *	0.780	4.08	66.29	50.02	3012.95
[bmim][BF_4_]/water *	0.544	13.00	96.88	89.42	4320.04
[emim][BF_4_]/water *	0.525	18.20	98.56	93.14	4476.38
[emim][C_2_H_5_SO_4_]/water *	0.569	13.57	97.92	90.70	4130.23
[mmim][(CH_3_)_2_PO_4_]/water *	0.662	5.32	93.66	76.06	3550.00
[bmim][I]/water *	0.534	23.70	98.75	94.58	4400.94
[choline][Gly]/water *	0.446	4.79	93.22	73.76	5269.28
[choline][CH_3_SO_3_]/water *	0.636	7.32	94.78	81.83	3695.13
[choline][Lac]/water *	0.659	7.79	96.71	84.29	3566.16
[bmim][(C_4_H_9_)_2_PO_4_]/water *	0.532	11.17	91.82	83.6	4417.48
[eeim][(C_2_H_5_)_2_PO_4_]/water *	0.565	12.38	95.99	88.24	4159.47
[emim][(C_2_H_5_)_2_PO_4_]/water *	0.588	7.75	90.28	78.63	3996.77
[emim][(CH_3_)_2_PO_4_]//water *	0.691	8.66	98.13	86.79	3401.01

* Reference [14].

**Table 5:** The temperature ranges considered in the experimental design analysis for the evaporator, regenerator, absorber, and condenser.

Temperature	Min (°C)	Max (°C)
T_evaporator_	5	15
T_regenerator_	60	150
T_absorber_	20	50
T_condenser_	20	50

**Table 6:** The standard deviations and R-Squared values of experimental design analyses for each of the DES/water working pairs.

Working Pair	Standard Deviation	R-Squared Value	Mean Value
Reline/water	0.0048	0.9978	0.64
Ethaline/water	0.0126	0.9454	0.68
Glyceline/water	0.0013	0.9996	0.67

**Table 7:** The optimum value of COP for each of the absorption refrigeration cycles and the corresponding optimized conditions.

Working Pair	Optimum Value of COP	*f*	*T_eva_* (°C)	*T_gen_* (°C)	*T_abs_* (°C)	*T_con_* (°C)	*Q_e_* (kW)	*Q_g_* (kW)
Reline/water	0.816	244.3	10.00	60.00	20.00	40.00	2150.9	2635.91
Ethaline/water	0.776	30.8	10.00	60.00	20.00	40.00	2150.9	2771.78
Glyceline/water	0.786	47.9	10.00	60.00	20.00	40.00	2150.9	2736.51

**Table 8:** Comparison between the work done by the pump and the heat transferred in the regenerator at the base case conditions.

Working Pair	*W_P_ (W)*	Heat ratio	*W_P_*/Q_g_ (%)
Reline/water	175.82	0.70	0.006
Ethaline/water	64.87	0.71	0.002
Glyceline/water	85.17	0.72	0.003

## Appendix A

The parameters of the ideal gas heat capacities of water and the investigated DESs are presented in Table A1:

**Table A1:** The values of the ideal gas heat capacity constants for water [35] and the investigated DESs in this study using the Joback group contribution method [34].

Substance		$A(\frac{J}{mol\cdot K})$	$B(\frac{J}{mol\cdot K^{2}})$	$10^{5}C(\frac{J}{mol\cdot K^{3}})$	$10^{7}D(\frac{J}{mol\cdot K^{4}})$
Reline		30.43067	0.275520	−6.4000	−4.400
Ethaline		23.31867	0.320320	−9.5000	−4.300
Glyceline		25.11867	0.410253	−15.0000	−4.100
Water		32.24685	0.001928	1.0557	−0.036

(A1) $$C_{p_{i}}^{ig}=A_{i}+B_{i}T+C_{i}T^{2}+D_{i}T^{3}$$

where *T* is in Kelvin and *Cp* is in $\left(\frac{J}{mol\cdot K}\right)$.

(A2) $$A_{DES}=x_{HBA}A_{HBA}+x_{HBD}A_{HBD}$$

(A3) $$B_{DES}=x_{HBA}B_{HBA}+x_{HBD}B_{HBD}$$

(A4) $$C_{DES}=x_{HBA}C_{HBA}+x_{HBD}C_{HBD}$$

(A5) $$D_{DES}=x_{HBA}D_{HBA}+x_{HBD}D_{HBD}$$

## Appendix B

The operational conditions of the DES-rich stream and the DES-lean stream are presented in Table A2 and Table A3, respectively, at the base case conditions.

**Table A2:** Temperature, pressure, and DES concentration in the DES-rich stream (stream 5) at the base case conditions for the three investigated working pairs.

Working Pair	Temperature	Pressure (kPa)	*x_5_* (Mass%)
Reline/water	322.87	1.227	97.46
Ethaline/water	328.03	1.227	92.38
Glyceline/water	326.32	1.227	94.50

**Table A3:** Temperature, pressure, and DES concentration in the DES-lean stream (stream 2) at the base case conditions for the three investigated working pairs.

Working Pair	Temperature (K)	Pressure (kPa)	*x_2_* (Mass%)
Reline/water	303.15	1.227	94.57
Ethaline/water	303.15	1.227	84.37
Glyceline/water	303.15	1.227	88.61
